# A systematic review and meta‐analysis of nonpharmacological interventions for children and adolescents with selective mutism

**DOI:** 10.1002/jcv2.12166

**Published:** 2023-05-03

**Authors:** Gino Hipolito, Emma Pagnamenta, Helen Stacey, Emily Wright, Victoria Joffe, Kou Murayama, Cathy Creswell

**Affiliations:** ^1^ School of Psychology and Clinical Language Sciences University of Reading Reading UK; ^2^ Paediatric Speech and Language Therapy Department St George's University Hospitals NHS Foundation Trust London UK; ^3^ Department of Experimental Psychology University of Oxford Oxford UK; ^4^ School of Health and Social Care University of Essex Colchester UK; ^5^ Hector Research Institute of Education Sciences and Psychology University of Tübingen Tübingen Germany; ^6^ Department of Psychiatry University of Oxford Oxford UK

**Keywords:** anxiety, intervention, outcomes, selective mutism, speaking behaviour, systematic review

## Abstract

**Background:**

Selective mutism (SM) is an anxiety disorder that often starts in early years with serious and lasting consequences. Nonpharmacological interventions are commonly seen as the preferred first treatment. This systematic review identifies outcome measures used and outcomes achieved for nonpharmacological interventions for children and adolescents with SM.

**Methods:**

Systematic searches were conducted using 13 electronic databases and hand searches, including peer‐reviewed and grey literature since 1992.

**Results:**

Twenty‐five studies were identified. While specific measures varied, all studies reported an outcome measure for speaking behaviour and 18 used a measure of anxiety. Few studies reported measures of SM remission (*k* = 6), well‐being (*k* = 6), academic impact (*k* = 2), or quality of life (*k* = 1). Within subject outcomes for nonpharmacological interventions were variable for improvements in speaking behaviours (very small to large positive effects) and reduction in anxiety symptoms (very small negative to large positive effects). Only five randomised controlled trials (RCTs) were included in the meta‐analysis. Three studies compared a combined systems/behavioural approach with waitlist controls indicating a significant and large effect (Hedges *g* = 1.06, *p* < .0001, 95% CI: 0.57–1.56) on improved speaking behaviour. Two of these RCTs showed a large effect for SM remission favouring the intervention (Risk Ratio = 4.25, *p* = .1774, 95% CI: 0.52–34.84) but this did not reach statistical significance. Non‐significant outcomes for two RCTs with active controls (Hedges *g* = 0.55, *p* < .2885, 95% CI: −0.47 to 1.57) showed considerable heterogeneity in approach and outcomes, one with large and one with negligible effects.

**Conclusion:**

Despite the considerable impairment caused by SM, there has been little systematic evaluation of non‐pharmacological interventions. Although combined systems/behavioural interventions are promising, further systematic evaluations are urgently needed to inform treatment approaches. Cross‐study measurement harmonisation is required to promote learning from all studies, including wider clinical and economic outcomes.

**Clinical Trial Registration:**

Not applicable.

## Graphical abstract

This broad systematic review found an improvement in study designs and manualised treatments in the selective mutism (SM) literature; however, few fully powered experimental studies, inconsistent use of measures, sampling gaps, insufficient reporting, and absence of health economic analyses prevent definitive answers to what works for these children and young people. Outcomes for SM remission and improving speaking behaviour for children (3–9 years) appear promising when using a combined systems and behavioural intervention approach.

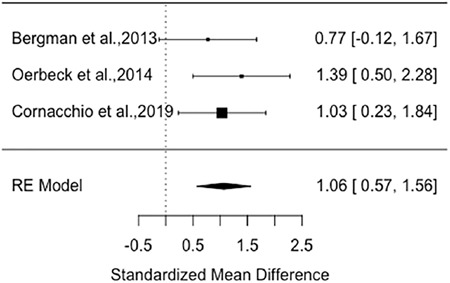

**Key points**

- This broad systematic review found an improvement in study designs and manualised treatments in the SM literature; however, few fully powered experimental studies, inconsistent use of measures, sampling gaps, insufficient reporting, and absence of health economic analyses prevent definitive answers to what works for these children and young people.
- Outcomes for SM remission and improving speaking behaviour for children (3–9 years) appear promising when using a combined systems and behavioural intervention approach.
- Future treatment studies are recommended to consistently report on intervention components, co‐morbidity, onset, duration, and severity of SM.
- Regional and international research collaborations and an agreed measurement harmonisation for SM treatment studies are needed to enable more fully powered experimental studies to improve the evidence base.

## INTRODUCTION

Selective mutism (SM) is a condition in which a person is unable to speak in certain situations where speech is expected (e.g. school or in public) but is able to speak in other situations (e.g. home) (DSM5; American Psychiatric Associations, 2013). Selective mutism is an anxiety disorder that often starts between the ages of 2 and 5 years with a prevalence of 1 in 140 children under 8 years of age (Bergman et al., 2002; Elizur & Perednik, 2003). It has serious consequences during childhood and poor long‐term outcomes. The inability to talk can have a negative impact on the child's education and social interactions with others in the short term (Bergman et al., 2002; Kumpulainen et al., 1998). There is also a high rate of emotional and behavioural problems in this group of children (Kristensen, 2001; Steinhausen & Juzi, 1996). Furthermore, two long‐term follow up studies (Remschmidt et al., 2001; Steinhausen et al., 2006) assessed adults who accessed a range of interventions for SM in childhood (average age of 8.5 years) and found 42%–61% continued to have SM in adulthood hindering their communication in education, leisure activities, and at work, with many continuing to have emotional and behaviour problems. Clearly, there is a need for effective interventions to address SM and mitigate the short‐ and long‐term negative effects of the condition.

To understand which interventions are effective, it is crucial for studies to use appropriate outcomes measures specific to the condition and that capture the impact and change of the disorder (Coster, 2013). In the first systematic review of SM interventions, Stone et al. (2002) found that ‘most’ studies used a narrow range of outcome measures, particularly focussed on speaking behaviour. In a more recent systematic review of assessment tools to screen and diagnose the core symptomology of SM, Rodrigues Pereira et al. (2021) found that the clinician administered Anxiety Disorders Interview Schedule (ADIS, Silverman & Albano, 1996) was most often used for diagnosing SM and comorbid anxiety disorders (*k* = 23), while the parent reported SM Questionnaire (Bergman et al., 2008) (*k* = 32) and teacher reported School Speech Questionnaire (Bergman et al., 2002) (*k* = 11) were the most common speaking behaviour outcomes in SM studies (*k* = 56) using a standardised measure in the last 10 years. However, overall there was a lack of consistency in the specific measures used for SM. This lack of consistency is a problem, as highlighted by Creswell et al. (2021), with reference to the general paediatric anxiety disorders literature, who argued that wide variation in outcome measures makes it difficult to compare and combine studies and risks faulty conclusions from meta‐analyses. Furthermore, Stone et al. (2002) raised concerns that few intervention studies report on the *impact* of SM on children or the wider implications, for example, educational outcomes; and no recent systematic reviews have investigated these outcomes. As a first step it is important to capture the range of measures used across interventions studies for SM in order to make recommendations to promote greater consistency going forwards.

In terms of treatment outcomes, although there is some evidence for efficacy of medication (specifically SSRIs) for children with SM, they are not typically seen as a first line treatment due to parental concerns about potential pharmacological side effects, particularly among young children (Manassis et al., 2016; Østergaard, 2018). On the other hand, nonpharmacological interventions, such as behavioural therapy, are perceived as effective and acceptable by parents of young children with SM (Bergman et al., 2013). Narrative reviews in the last 30 years have described nonpharmacological interventions and their outcomes (Anstendig, 1998; Cohan et al., 2006; Viana et al., 2009; Zakszeski & DuPaul, 2017) but have not systematically investigated effectiveness. Stone et al. (2002) provided the first synthesis of SM intervention research and showed that behaviour therapy was more effective in improving speaking behaviour than no treatment with a median effect size (standardised mean difference) of 1.63. However, the meta‐analysis consisted of single case design studies only with small samples of up to 4 participants and so was limited to within group effect sizes which carry a high risk of bias. At that point in time, there were no randomised controlled trials (RCTs) in SM intervention research. Advances in SM research have led to larger studies and RCTs, prompting Steains et al. (2021) more recently to carry out a systematic review and meta‐analysis of five RCTs. They found that psychological interventions were more effective than no treatment with a large treatment effect (overall weighted effect size Hedges' *g* = 0.87). However, this effect size combined a broad range of outcomes including speaking behaviour, SM severity rating, anxiety, global functioning and social anxiety severity. Although a sensitivity analysis found that the means did not differ significantly for SM specific compared to non‐SM specific outcomes, there was extensive heterogeneity within these two categories of outcomes. Consequently, it is not clear what the effect sizes were for the specific outcomes and whether they differed according to type of intervention. Moreover, no systematic review has investigated the rate of SM remission after nonpharmacological intervention further limiting our understanding of effectiveness.

The aim of this paper is to carry out a broad systematic review of nonpharmacological interventions for children and adolescents with SM. The systematic review will address the following questions:

1. What are the outcome measures used in nonpharmacological intervention studies for children and adolescents with SM?
2. What are the outcomes and remission rates in nonpharmacological interventions for children and adolescents with SM?
3. Which nonpharmacological interventions for children and adolescents are effective in increasing SM remission and improving speaking behaviour (the primary symptom of SM)?

## METHODOLOGY

This review followed the Preferred Reporting Items for Systematic Reviews and Meta‐Analyses (PRISMA) guidelines (Moher et al., 2009) and was registered on the PROSPERO International prospective register of systematic reviews on the 2 September 2019, registration number CRD42019147573. As we anticipated that there would be only a small number of RCTs, we opted to conduct a broad review including both RCTs and non‐RCTs (including grey literature) to reduce publication bias and provide a comprehensive and balanced view of the evidence (Paez, 2017).

Searches in 13 databases were initially carried out between 20 September and 14 October 2019. Searches were replicated on the 29 January 2021 and 7 January 2022 to add further relevant studies. Double coding was used for the screening, full text eligibility and quality appraisal sections of the review with any disagreements resolved by an independent reviewer. Details of the search strategy, eligibility criteria, data extraction, selection procedures, data management and quality appraisal are given in Appendix S1 and Table S1.

### Strategy for data synthesis

Due to the anticipated range of study designs in this broad review and to allow for comparison in the narrative synthesis, within case effect sizes of the intervention were calculated in each study using Hedges g standardised mean differences, which is useful for bias correction in small sample sizes (Borenstein et al., 2009), for continuous outcome variables. The Hedges g within case effect size was calculated for pre and post data using the open source ‘effsize’ package (Torchiano, 2020) in R if the raw data was available and a Hedges' g* unbiased formula (Borenstein et al., 2009) in Microsoft Excel (2016) if only the pre and post mean and standard deviation were available. In the latter situation, a pretest‐posttest correlation of 0.5 has been imputed in the formula. The direction of the within case effect sizes was corrected to be in line with the between subject effect sizes for comparability.

The Hedges g between subject effect size (Hedges et al., 2013) was calculated for multiple baseline single case experimental design using the ‘scdhlm’ package (Pustejovsky et al., 2020) in R to be in the same metric to compare with RCTs (Shadish et al., 2014). The Hedges g between subject effect size for RCTs were calculated using the ‘esc’ Package (Ludecke, 2019) in R. Positive values in effect sizes for speaking behaviour indicated that participants in the treatment group improved (relative to the control group where applicable). Negative values in effect sizes for anxiety measures indicated that the treatment group had lower anxiety levels after treatment (in comparison to the control group where applicable).

To measure the effectiveness of the interventions, random effects meta‐analyses were conducted using ‘metafor’ package (Viechtbauer, 2010) in R where there were two or more eligible RCTs (Valentine et al., 2010). The speaking behaviour outcomes (primary SM symptom) were calculated as Hedges' g standardised mean difference while SM remission outcomes were converted into risk ratios. The Q statistic was used as a test of significance of heterogeneity (Borenstein et al., 2009), while the I^2^ index evaluated the extent of the heterogeneity between studies (Deeks et al., 2008).

## RESULTS

**FIGURE 1:**
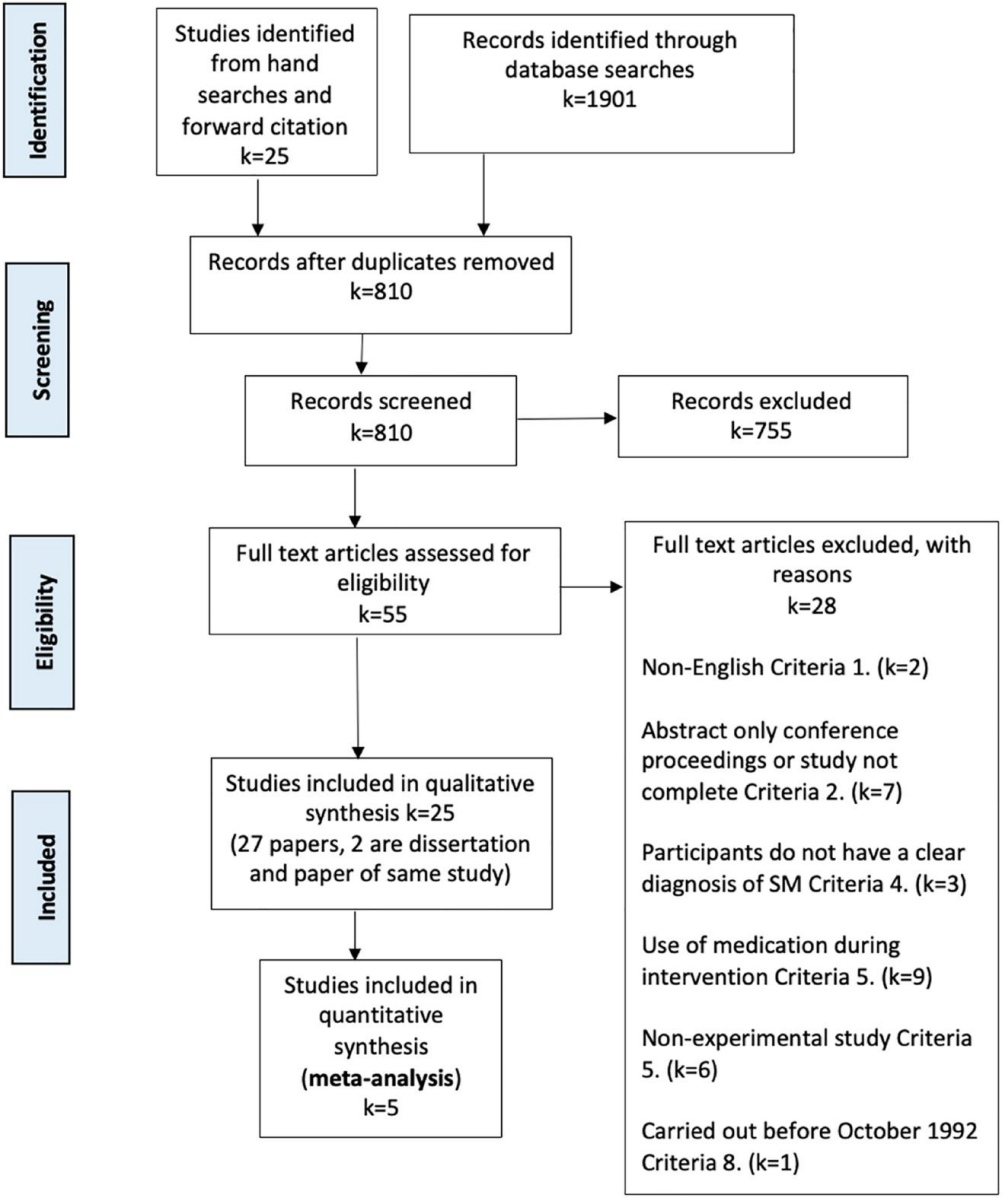
PRISMA flowchart.

The search strategy yielded 810 references after duplicates and pre‐1992 studies were removed. The references were screened by title and abstract with exclusion codes recorded. The reasons for exclusion at this stage were due to: non‐intervention study; not specific to SM; *n* < 3; pharmacological study; age >18 years or non‐English abstract. Fifty‐five papers remained for the full text eligibility assessment, of which a further 28 were excluded (see Figure 1). Twenty‐five studies (described in 27 papers) were included in the qualitative synthesis, five of which underwent quantitative synthesis. Complete data of Selective Mutism Questionnaire (SMQ) scores were requested and received from Oerbeck et al. (2014) and Cornacchio et al. (2019). Characteristics of the included studies are provided in Table 1, organised by study design, of which six were RCTs (24%), five were single case experimental designs (20%), eight were pre‐experimental one group pretest posttest design (32%) and six were pre‐experimental single subject designs (24%).

**TABLE 1:** Study characteristics.

ID	Authors	Study design	Participants							Intervention				
			*N*	Gender (male)	Age range	Average onset of SM (years)	Average duration of SM (years)	Bi or multi‐lingual	Co‐morbidity	Type of Tx (broad)	Type of Tx (specific)	Who provided Tx	Location of Tx	Dosage of Tx (hours)
Experimental: Randomised control trial (RCT) versus active control														
1	Ooi et al. (2016)	RCT	21	8	6–12	NR	NR	NR	5 SP, 2 SeAD, 2 SAD	BT	Online CBT versus computer games	Clinician	Clinic	14 weekly sessions (14)
2	Esposito et al. (2017)	RCT	166	90	6–18	NR	NR	NR	NR	BSBoT	Psychomotor therapy versus Behavioural & educational counselling	Clinician, parent	Home	24 weeks, 45 min sessions ×3 weeks (54)
Experimental: RCT versus waiting list (WL)														
3	Bergman et al. (2013)	RCT	21	11	4–8	3.38	2.05	NR	18 SAD	BST	Integrated behaviour therapy for SM (IBTSM)	Clinician, parent, teacher	Clinic, public places, school	24 weeks, 20 sessions (20)
4	Oerbeck et al. (2014)	RCT	24	8	3–9	NR	NR	6	15 MD or LaD, 24 SAD, 7 SeAD, 6 SP, 2 GAD, 2 OCD, 2 Tics, 6 Enu, 1 Enc	BST	Home and school CBT	Clinician, parent, teacher	Home, school	12 weeks, 21 sessions (12)
5	Cornacchio et al. (2019)	RCT	29	7	5–9	NR	NR	NR	21 SAD, 8 SeAD, 7 GAD, 3 SP, 2 OCD, 2 Enu, 2 ADHD	BST	Intensive group behavioural treatment (IGBT) based on PCIT‐SM	Clinician or Trained volunteer, parent	Simulated classroom, camp, public places	1 week, 5 days of 6–8 h (35)
Experimental: RCT comparing adapted treatments														
6	Bunnell et al. (2018)	RCT	15	5	5–17	NR	NR	NR	15 SAD, 2 SeAD, 1 GAD, 2 Enu	BT	Shaping with apps versus Shaping with other tools versus Shaping with reinforcement only	Clinician	Clinic	2 sessions of up to 55 min (2)
Experimental: Single case experimental design (SCED)														
7	Stone (2000)	SCED (matched pair)	6	2	4–9	2.66	3.16	NR	1 Enu, 1 LaD	BST	Videotape Training (VT) versus Video self modelling (VSM)	Parent, teacher guided by clinician	School	Both Txs: 12 weeks, 12–24 sessions, VT (27.5), VSM (20)
8	Vecchio (2008); Vecchio and Kearney (2009)	SCED (alternating treatment design)	9	2	4–9	Not clear (range 2–5)	Not clear (range 2–4)	NR	9 SAD, 2 SeAD, 2 SP, 1 ADHD, 1 Enu, 1 GAD, 1 ODD, 2 SpD	BT	Exposure practices and contingency management	Clinician, parent	Clinic, home, public places, school	8–20 weeks, 2 sessions a week (NR)
9	Mitchell and Kratochwill (2013)	SCED (multiple baseline)	4	2	5–10	NR	3	NR	NR	BST	Conjoint behavioural consultation	Parent, teacher guided by clinician	Clinic, school	10 weeks, 3–7 sessions, (NR)
10	Solz (2015)	SCED (multiple baseline)	3	1	5–9	NR	NR	NR	1 SAD, 1 ASD, 1 SpD	BST	Video self‐modeling with and without contingency management	Clinician, parent, teacher	Clinic, school, home	3–4 weeks, 12 sessions (2)
11	Siroky (2019)	SCED (multiple baseline)	5	3	4–8	NR	NR	0	3 SAD, 2 GAD, 1 SeAD	BST	Condensed IBTSM	Clinician, parent	Clinic	16–22 weeks, 16 sessions, (13)
Pre‐experimental: One‐group pretest posttest design														
12	Woodcock et al. (2007)	One‐group pre‐post test design	15	6	4–8	2.5	3.3	7	7 SAD, 3 SeAD, 2 GAD	BST	School based CBT	Parent, teacher guided by clinician	School, public places	12 months, 12 training sessions, parents & teachers 20 min × 3 weeks for 6 months (24)
13	Sharkey et al. (2008)	One‐group pre‐post test design	5	1	5–8	3.6	2.4	NR	1 SeAD	BST	Parent group (systems) and child group therapy (CBT)	Clinician, parent	Clinic, with homework in public places and school	8 weeks, 8 sessions (12)
14	Oerbeck et al. (2011)	One‐group pre‐post test design	7	2	3–5	NR	1.6	4	1 LaD	BST	Home and school CBT	Clinician, parent, teacher	Home, school	14 weeks, (12)
15	Oerbeck et al. (2015)	One‐group pre‐post test design	24	8	3–9	NR	NR	6	15 MD or LaD, 24 SAD, 7 SeAD, 6 SP, 2 GAD, 2 OCD, 2 Tics, 6 Enu, 1 Enc	BST	Home and school CBT	Clinician, parent, teacher	Home, school	12 weeks, 21 sessions (12)
16	Klein et al. (2017)	One‐group pre‐post test design	40	NR	5–12	2.81	3.96	NR	27 LaD	BST	Social communication anxiety treatment (S‐CAT)	Clinician, parent, teacher	Clinic, school, public places	9 weeks, 3 sessions every 3 weeks, (NR)
17	Oerbeck et al. (2018)	One‐group pre‐post test design	32	10	3–9	NR	NR	9	16 MD or LaD, 24 SAD, 7 SeAD, 6 SP, 2 GAD, 2 OCD, 2 Tics, 6 Enu, 1 Enc	BST	Home and school CBT	Clinician, parent, teacher	Home, school	12 weeks, 21 sessions (12)
18	Aldrich et al. (2021)	One‐group pre‐post test design	112	40, 1 trans male	3–14	NR	NR	35	NR	BST	Multi‐disciplinary SM treatment group based on PCIT‐SM	Clinician	Clinic, locations in hospital	8 weeks, 8 sessions (12)
19	Tan et al. (2021)	One‐group pre‐post test design	24	11	6–12	NR	NR	NR	7 SAD, 7SP, 3 SeAD, 1 GAD	BT	Virtual reality exposure therapy (VRET)	Clinician	Clinic, virtual classroom	10 weeks, 6 sessions (6)
Pre‐experimental: Single subject design														
20	Ortega (2011)	ABC	4	2	6–7	NR	NR	NR	NR	BST	Totally anxiety‐free communication treatment (CBT)	Clinician, parent, teacher	Clinic, school	18‐22 sessions, 2–3 sessions a week (NR)
21	Paasivirta (2012)	ABA	4	1	5–7	NR	NR	2	3 AD‐NOS, 1SP, 1 Enu, 1 Enc	BST	Brief teacher training programme	Teacher guided by clinician	School	3–4 weeks, 1 h training, 2–3 weekly sessions for 20 min, (2)
22	Roslin (2013)	AB	5	2	4–7	NR	NR	NR	2 SAD, 1 SP	BST	Parent child interaction therapy (PCIT‐SM)	Clinician, parent	Clinic, public places	12 weekly sessions, (12)
23	Bunnell et al. (2016)	AB	4	1	7–10	4.25	4	NR	NR	BT	Shaping with apps	Clinician	Clinic	2 sessions of up to 55 min (2)
24	Bork (2016); Bork and Bennett (2020)	AB	3	0	8	3.2	2.8	NR	NR	BT	Video self‐modeling and stimulus fading	Parent, teacher guided by clinician	School	8 weeks, 2 sessions a week, (1–2)
25	Haggerty (2020)	AB	25	5	4–11	NR	NR	0	20 SAD, 5 GAD	BST	Intensive summer day camp for SM	Clinician, parent	Simulated classroom, camp, public places	5 days, (40)

*Note*: AB refers to A (baseline) phase and the B (intervention) phase; while ABA refers to A (baseline) phase, B (intervention) phase, and A (withdrawal/follow up) phase; ABC refers to A (baseline) phase, B (intervention) phase, and C (other intervention) phase.

Abbreviations: ADHD, attention deficit hyperactivity disorder; AD‐NOS, anxiety disorder not otherwise specified; ASD, autism; BSBoT, behavioural systems body‐oriented therapy approach; BST, behavioural systems therapy approach; BT, behavioural therapy approach; Enc, encopresis; Enu, enuresis; GAD, generalised anxiety disorder; LaD, language difficulties; MD, motor delay; NR, not reported; OCD, obsessive compulsive disorder; ODD, oppositional defiant disorder; SeAD, separation anxiety disorder; SAD, social anxiety disorder; SP, specific phobia; SpD, speech difficulties; Tics, tic disorders; Trans, transgender.

### Quality appraisal

Of the 25 studies, eight (32%) were considered to be of high quality (low risk of bias) while the rest (68%) were rated as low quality (high risk of bias) (see Table S2). The weak aspects of the studies (see Table S3) related to the method of subject selection, lack of randomisation, lack of blinding, inappropriate sample sizes, analytic methods insufficiently described or justified, estimate of variance reported for main results and lack of control for confounding variable. Only 12 studies (48%) calculated and reported effect sizes of the intervention varying considerably from Cohen's *d* (*k* = 8), Reliable Change Index (*k* = 3), Partial eta squared (*k* = 2), Hedges g (*k* = 1), Percentage of All Non‐overlapping Data (*k* = 1), Kendall's W (*k* = 1), *r* (*k* = 1) to Tau‐U (*k* = 1).

### Participant characteristics

All of the studies (*k* = 25) included children aged 9 years or under. Only nine studies (35%) also involved children 10 years or older, out of which only three (12%) included teenagers. Regarding SM characteristics, seven studies reported the age of onset (ranging between 2 and 5 years) and nine reported the duration (ranging between 1 and 5.5 years) of the condition prior to receiving treatment. Seven studies (28%) reported the frequency of bilingualism or multilingualism (representing between 25% and 57% of the samples); 19 studies (76%) reported on comorbidities, with 16 listing anxiety disorders, eight speech and language delays or impairments, and nine other developmental/neurodevelopmental problems (e.g. motor delay, enuresis, attention deficit hyperactivity disorder).

### Types of interventions

**FIGURE 2:**
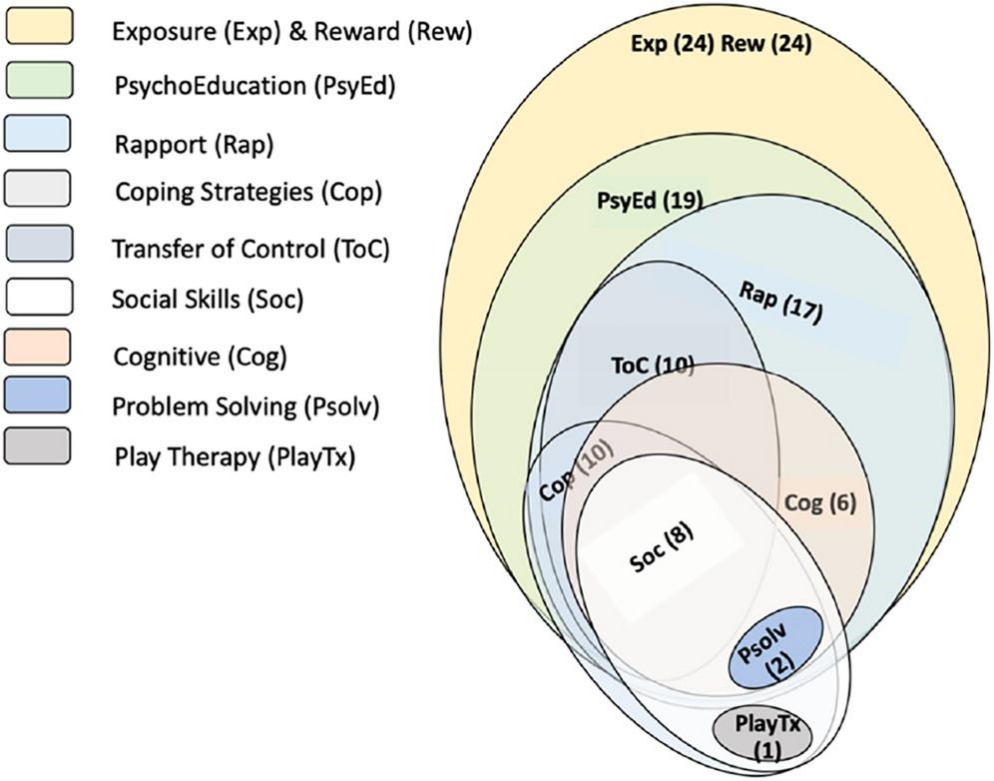
Treatment components in nonpharmacological selective mutism interventions.

Six studies (24%) considered behavioural therapy only. Eighteen studies (72%) used a combined behavioural and systems approach while one study (4%) incorporated a combined behavioural, systems and body‐oriented therapy closely linked to play therapy (psychomotor therapy). As shown in Figure 2 (and Table S4) the interventions varied in their use of components. The most common intervention components were exposure activities (*k* = 24) (e.g. graded exposure, stimulus fading, shaping, video self‐modelling, prompting strategies during speaking situations, see Zakszeski & DuPaul, 2017 for behavioural strategy definitions) and reward systems (*k* = 24) (e.g. contingency management, reinforcement, contingent and specific verbal praise) used in 96% of interventions, followed by psychoeducation for parents and/or teachers (*k* = 19, 76%) (e.g. understanding the condition, treatment, managing anxiety and maintenance of SM), and rapport building or adapting interaction style strategies (*k* = 17, 68%) (e.g. defocused communication, nonchalant communication, child‐directed interaction, verbal‐directed interaction). Less common intervention components focussed on coping strategies (*k* = 10, 40%) (e.g. relaxation, emotional regulation, breathing exercises), transfer of control (*k* = 10, 40%), social skills (*k* = 8, 32%), cognitive (*k* = 6, 24%) (e.g. cognitive restructuring), problem solving (*k* = 2, 8%) and play therapy (*k* = 1, 4%) (See Table S5 for component definitions).

### Characteristics of the interventions

Four studies (16%) delivered the intervention via a group format while the remaining studies (84%) took an individual approach. Twenty‐three studies (92%) had a protocol or manual for the intervention while two (8%) did not report any. Regarding who delivered the intervention with the child, the most common combination involved the clinician, parent, and teacher (*k* = 12, 48%). In four of these, the clinician guided the parent and teacher without directly working with the child. Seven studies (28%) involved both the clinician and parent, five studies (20%) used the clinician only, and one study involved the teacher only being guided by the clinician. The interventions were delivered in various combinations of locations including the school/simulated classroom, in a clinical setting, in the community, and/or the child's home. Four (16%) interventions were brief and/or intensive treatments lasting less than a week ranging between 2 and 40 h of therapist contact, eight (32%) were between 2 and 9 weeks with 2–12 h of therapist contact, ten (40%) lasted between 10 and 19 weeks varying between 12 and 27.5 h of therapist contact while three (12%) continued for 20 or more weeks requiring 20–54 h of the clinician's time.

### Outcomes assessed

A wide range of outcomes were used to assess speaking behaviours across the studies (See Table S6). While the SM Questionnaire (76%) and/or the School Speech Questionnaire (48%) were consistently used, 20 other outcome measures were employed by studies which varied in measurement mode (e.g. rating scales, counts or time), respondents (e.g. independent observers, clinician, teachers, parents or child), and type of communication (e.g. words spoken, whispered, mouthed, non‐verbal).

The ADIS for Children (ADIS‐C/P) was consistently used to measure SM remission for all six studies with this data, however outcomes were reported in different ways. For example, in two of the studies, the authors used three categories: ongoing SM, partial remission and full remission, that is, where children no longer fulfiled the diagnostic criteria *and* were speaking freely in school (Oerbeck et al., 2015, 2018). Five studies assessed comorbid remission of social anxiety disorder (SAD); three used the ADIS‐C/P and two used the Schedule for affective disorders and schizophrenia for school aged children (K‐SADS‐PL, Kaufman et al., 1997).

Eighteen studies (72%) measured anxiety symptoms with 18 different measures. Ten studies (40%) measured condition severity and/or improvement however, again these varied in measures used and timings of assessments (see Table S6). Only a few studies measured broader impacts of SM on the child’ life: well‐being (social, emotional and behavioural problems) (*k* = 6; 24%); functional impairment or interference (*k* = 4; 16%); communication progress (*k* = 2; 8%); academic impairment/competence (*k* = 2; 8%); and quality of life (*k* = 1, 4%). Three studies (12%) measured changes in adult interactions with the child.

### Outcomes

#### Within case effect sizes and percentage of remission

A detailed description of findings is provided in Appendix S2 and Table S3 for within case effect sizes for the study outcomes and percentage remission rates. Overall a mixed pattern of outcomes was found across the outcome variables. The few studies that reported remission rates (SM, *k* = 6; SAD, *k* = 5) displayed positive results. Most studies reported improvements in speaking behaviours (*k* = 21) and wider outcomes when reported such as well‐being (*k* = 3), functional impairment or interference (*k* = 4), and academic impairment (*k* = 2) with varying within case effect sizes from slight to large. Notably, for anxiety outcomes, some studies reported positive (*k* = 12), mixed (*k* = 4) and negative (*k* = 1) changes following intervention ranging between large effect sizes decreasing and small effect sizes increasing anxiety.

### Between subject effect size

#### Improvements in speaking behaviours in single case experimental designs

Mixed results were also found based on speaking behaviour and other communication outcomes in Single Case Experimental Designs. Mitchell and Kratochwill (2013) showed an increase in frequency of words spoken per minute with a large between subject effect size following a combined behavioural and systems approach (Hedges' *g* = 2.08, 95% CI: 0.78–3.38). In contrast, Solz (2015) found a negligible between subject effect size in verbalisations (Hedges' *g* = 0.05, 95% CI: −0.36 to 0.46), yet a large effect size in increased meaningful communication (Hedges' *g* = 1.46, 95% CI: 0.90–2.02) following a combined behavioural and systems approach. Siroky (2019) also found a small between subject effect size of observed speaking behaviours (Hedges' *g* = 0.36, 95% CI: −0.17 to 0.89) following a combined behavioural and systems approach.

### Meta‐analysis

**FIGURE 3:**
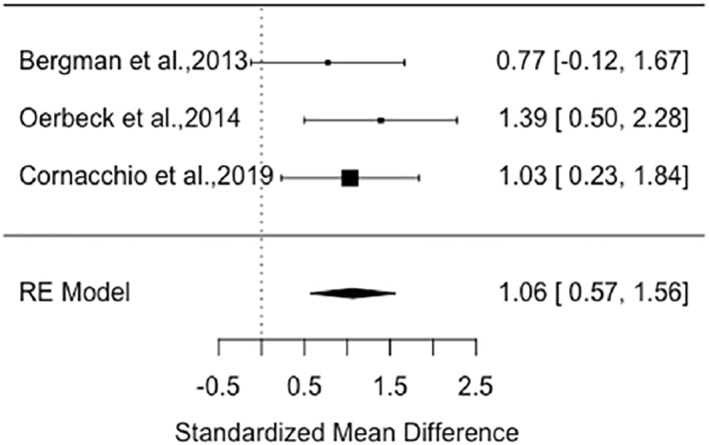
Speaking behaviour: hedges g forest plot for treatment versus waiting list control.

**FIGURE 4:**
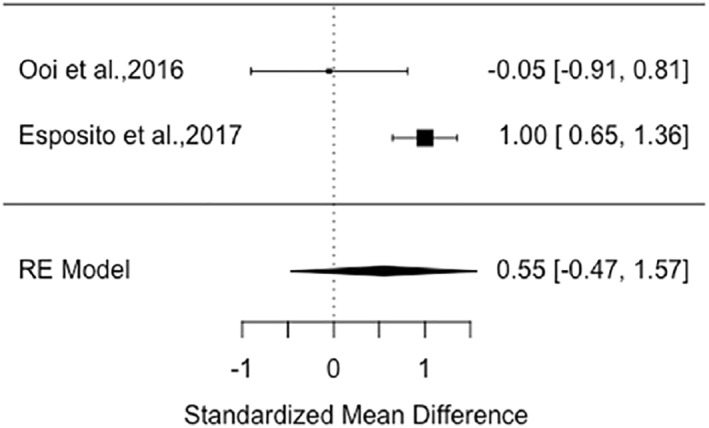
Speaking behaviour: hedges g forest plot for treatment versus active controls.

**FIGURE 5:**
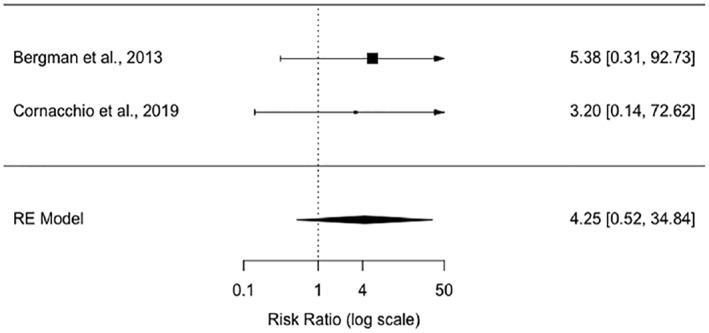
Selective mutism remission: risk ratio (log scale) forest plot for treatment versus waiting list control.

Five RCTs were suitable for meta‐analysis of change in speaking behaviour (all used the SMQ) of children after an intervention/control period displayed in two forest plots that differed in controls (see Figures 3 and 4). Two of these RCTs had appropriate data for a meta‐analysis of SM remission (ADIS) shown in a third forest plot (see Figure 5).

The first forest plot (see Figure 3) consisted of three nonpharmacological intervention studies involving 38 treatment participants compared to 36 waiting list (WL) control participants. Non‐pharmacological interventions had a large positive effect on speaking behaviour compared to WL controls (Hedges *g* = 1.06, *p* < .0001, 95% CI: 0.57–1.56). The results indicated that there was not significant variation between effect sizes (*Q* = 0.93, df = 2, *p* = .63) with minimal statistical heterogeneity (*I* ^2^ = 0.00%, τ^2^ = 0 with SE = 0.19) (Borenstein et al., 2009; Deeks et al., 2008). Due to the minimal heterogeneity and low number of studies a moderator analysis was not carried out. It is important to note that the effect size from Bergman et al. (2013) was calculated using the SMQ scores at week 12 in order to directly compare with the WL period, however it was only half‐way through the treatment so the effect size may have been greater by week 24 (12 weeks SMQ mean = 1.32, SD = 0.49; 24 weeks SMQ mean = 1.74, SD = 0.54).

The second forest plot (see Figure 4) comprised the two nonpharmacological intervention studies with a total of 88 treatment participants compared to 99 active control participants (computer games, behavioural and educational counselling to parents). There was a moderate sized improvement in speaking behaviour in the non‐pharmacological interventions compared to active controls, however this did not reach statistical significance (Hedges *g* = 0.55, *p* < .29, 95% CI: −0.47 to 1.57). The results indicated that there was significant variation between effect sizes (*Q* = 4.93, df = 1, *p* = .03) with considerable statistical heterogeneity (*I* ^2^ = 79.72%, τ^2^ = 0.44 with SE = 0.78) (Borenstein et al., 2009; Deeks et al., 2008), which reflects the disparity between Esposito et al.’s (2017) large treatment effect (Hedges *g* = 1.00, *p* < .001, 95% CI: 0.64–1.36) and Ooi et al.’s (2016) negligible treatment effect (Hedges *g* = −0.05, *p* < .994, 95% CI: −0.91 to 0.81). The clinical heterogeneity may also explain the different results between these two studies with contrasting intervention approach, active control, dosage, location, and provider of the intervention (see Table 1). Due to the small number of studies, differences in control conditions, and the varying effects, it is difficult to know what the true dispersion (τ^2^) looks like (Borenstein et al., 2009). Therefore, these results must be treated with caution.

The third forest plot (see Figure 5) incorporate two nonpharmacological intervention studies (Bergman et al., 2013; Cornacchio et al., 2019) with a total of 26 treatment participants compared to 24 control participants. There was a large effect for SM remission favouring the intervention; however, this did not reach statistical significance due to the small number of studies and participants (Risk Ratio = 4.25, *p* = .18, 95% CI: 0.52–34.84). The results indicate that there was no significant variation between effect sizes (*Q* = 0.06, df = 1, *p* = .81) with minimal statistical heterogeneity (*I* ^2^ = 0.00%, τ^2^ = 0 with SE = 3.29). However, due to the small number of studies these results must be treated with caution.

## DISCUSSION

This systematic review identified 25 studies that evaluated nonpharmacological interventions for children and adolescents with SM. Although there has been progress since Stone et al. (2002) systematic review, with notably more experimental studies (44%) and manualised treatments (91%), only six RCTs have systematically tested nonpharmacological interventions for SM which together included only 276 children. Five of the six RCTs were suitable for meta‐analysis, and the outcomes suggested that combined systems/behavioural approaches are promising treatments for helping children (3–9 years) with SM speak to more people in the home, school and community. A meta‐analysis of two of the RCTs also suggested that combined systems/behavioural interventions have promising outcomes in terms of SM remission. However, overall, the few systematic evaluations limit conclusions about the effectiveness of nonpharmacological interventions.

Only six studies measured actual remission of SM, and these had different assessment timings and classification of remission. This is likely to be an important outcome given it takes into account both symptoms and impairment, which have been highlighted as meaningful to families in the wider child anxiety literature (Creswell et al., 2021). Furthermore, of these six studies, only two were RCTs. Although, the results indicated a large effect for SM remission, favouring the intervention (RR = 4.5), the small number of studies and participants meant the results lacked precision and were not statistically significant leading to cautious conclusions. An agreed standard approach to reporting SM remission outcomes would help with consistency and enable meaningful comparisons in future analyses.

The lack of consistency of measures used also means that, at best, only tentative conclusions about outcomes for children and adolescents with SM can be drawn. The most consistent measure for speaking behaviour was the SM Questionnaire used in all of the RCTs included in the meta‐analyses. However, the single case design studies measured speaking behaviours in broader ways (including words spoken, whispered, mouthed, non‐verbal communication), with varying lengths of time (per session, per day, per minute) from different assessors (the clinician, parent, teacher, or child). Consequently, there is limited meaningful comparison for speaking behaviour outcomes between the identified Single Case Experimental Designs and RCTs.

While the SMQ has played a significant part in international SM research (Rodrigues Pereira et al., 2021), including the RCTs in this systematic review and meta‐analysis, there are still some gaps in its utility which need to be addressed before it is recommended as the speaking behaviour outcome for SM intervention research with children and adolescents. There are many positives in using this measure. It is standardised and psychometrically sound (Cronbach's *α* = .97); widely used; adaptable, having been translated in Norwegian (Oerbeck et al., 2014), Italian (Esposito et al., 2017), Spanish (Olivares‐Olivares et al., 2021), Dutch (Rodrigues Pereira et al., 2022) and Japanese (Kakuta et al., 2022) and also into a teacher (School Speech Questionnaire, Bergman et al., 2002) and child reported measure (SMQ‐C, Milic et al., 2020). However, the SMQ's psychometric properties have only been validated for 3‐ to 11‐year‐olds limiting its use for the adolescent population. As suggested by Bergman et al. (2008) themselves, there is a need for further research to establish whether the SMQ is suitable for adolescents or needs to be adapted. Rodrigues Pereira et al. (2022) has started that process by including adolescents in their sample (3–17 years) when validating the Dutch translation. An alternative outcome measure has been developed that covers the age ranges of 3–18 years, the Frankfurt Scale of Selective Mutism (FSSM, Gensthaler et al., 2020) with promising psychometric properties in both a diagnostic scale (Cronbach's *α* = .90) and severity scale (Cronbach's *α* = .98). It is a parent reported questionnaire, similar to the SMQ, which could be useful for future SM intervention research due to its age range and dual function (diagnostic and symptom severity outcome). This may also facilitate improved reporting of remission rates.

From the 18 studies that measured anxiety outcomes, the within subject effects were highly variable, from small negative to large positive effects with no clear pattern based on the type of treatment or who rated anxiety (child, parent, or teacher). Only one study used objective physiological anxiety measures (Bunnell et al., 2018). The multifarious ways in which anxiety was measured, and the different timings of measurements, may have contributed to the heterogenous results. Again, there is a need for consensus on which anxiety outcomes to include in SM intervention research to promote consistency for future analyses. A possible starting point for this conversation could consider the wider guidelines for assessment in treatment trials for anxiety disorders more generally (Creswell et al., 2021). In these guidelines, the authors made recommendations on the reporting of diagnostic outcomes, symptom‐based and functional interference measures along with sample and treatment characteristics. More specific to anxiety outcomes, the authors recommended researchers consistently include: a multidimensional measure of anxiety symptoms, a psychometrically reliable and valid measure of the targeted symptoms, and an interference/impact measure.

Few studies measured the broader impact of the intervention on the child's life. This is concerning given the social, educational and vocational impairment associated with SM. The importance of capturing these impacts alongside remission of SM is highlighted by Remschmidt et al.’s (2001) long‐term follow up study in which young people who no longer had SM 12 years post‐intervention continued to struggle with independence, academic achievement, confidence and health when compared to a non‐SM reference group. Although, notably, Oerbeck et al.’s (2018) longitudinal study suggested that children and adolescents with SM had comparable quality of life to other Norwegian children 5 years after the combined systems and behavioural intervention. Two decades after Stone et al.’s (2002) initial systematic review there is still scarce evidence of changes in the broader impact of SM after intervention.

Besides the overall lack of available research, limitations in the quality of the evidence, variable intervention approaches, sampling gaps, insufficient reporting, and absence of health economic analyses in the current intervention literature all prevent definitive answers to questions about what works for children with SM. Indeed the majority of studies were rated as low quality indicating a high risk of bias. The main areas of weakness were the lack of randomisation, lack of blinding, small sample sizes and lack of control for confounding results either via study design (e.g. randomised controlled trial) or through statistical methods (e.g. partial correlation). These areas of weakness reflect the few sufficiently powered RCTs and lack of collaborative work across regions to enable larger samples sizes in SM intervention research. By pooling resources, there will also be greater opportunities to ensure that treatment outcome studies are generalisable to children with SM with more diverse backgrounds and characteristics. Of particular note, only three studies included adolescents 13 years and above which may be impacted by the lack of appropriate speaking behaviour outcome measures for this age range. Consequently, the results in this review are most applicable to children (3–9 years) with SM. Furthermore few studies included or reported on bilingual children, comorbid communication disorders (e.g. speech disorders, stammers, language disorders) and neurodevelopmental conditions (e.g. autism). This is concerning since these are all common among children with SM (Kristensen, 2000; Muris & Ollendick, 2021; Steffenberg et al., 2018; Toppelberg et al., 2005). Few studies reported variables such as participants' age of onset and duration of SM (Stone et al., 2002) and severity and familial SM (Remschmidt et al., 2001) which are associated with persistent long‐term SM. It will be critical that future studies consistently report these variables as potential moderators to understand the impact they may have on treatment outcomes. Going forward it will also be important to evaluate interventions in the settings in which they are routinely applied. Only just over half of the studies administered the intervention in routine clinical settings or in schools. Although there is one notable example of a research team that documented progress of their SM intervention from development (Oerbeck et al., 2011) to feasibility/pre‐evaluation (Oerbeck et al., 2014) to implementation (Oerbeck et al., 2015, 2018), finding large treatment effects on children's speaking behaviour when delivered by local therapists in community health clinics (Oerbeck et al., 2014).

Currently in SM intervention research, it can be difficult to blindly assess the treatment effects since most of the outcomes are based on parent or teacher report who are both often involved in the intervention. A potential solution to this problem is the use of an objective outcome measure for speaking behaviour. One example, that has come from recent advances in wearable technology, is a passive audio vocal measurement that appears to have the capabilities to quantify child vocalisations, vocal volume, and other conversational measures (Xu et al., 2018). While wearable technology is still developing and refining, it has potential to significantly improve the quality of SM research in the future.

In the future, it will be important for the field to explore wider questions about what works for whom, how, and overall economic viability. A current barrier to this includes the wide variation in intervention approaches, with limited descriptions of the components delivered within these interventions. As a first step we have proposed a set of definitions that can guide consistent reporting of treatment components going forward (see Table S5). The absence of health economic analyses in the literature limits our understanding of which SM interventions are worth investing in (Coast et al., 2018). Indeed, the wide variability in the amount of clinician time across the RCT interventions (from 12 to 54 h of clinician time) is likely to substantially influence their cost effectiveness, as are changes in wider health and societal costs, such as disruption to education, employment, use of health and social care services.

Strengths of this systematic review include the broad approach. The inclusion of grey literature minimises selection bias and provides a comprehensive picture of the research landscape. Robust procedures were followed, including the use of double raters for screening, full text eligibility and quality appraisal. Where possible we also conducted separate meta‐analyses for distinct outcomes (i.e. speaking behaviour improvement and SM remission). However, several limitations should also be noted. Firstly, the review only included studies written in English which risks the exclusion of relevant studies. Secondly, due to few trials, the effect sizes of all the studies' outcomes were converted into the within case metric for comparison which poses a risk of over inflation or an artefact of regression to the mean. Thirdly, since the review focussed on nonpharmacological interventions, studies with participants taking medication and where they did not explicitly state they had been on a stable dose for at least 1 month prior to and during the intervention were excluded. Consequently, nine potentially relevant studies were not included.

In conclusion, the outcomes for SM remission and improving speaking behaviour for children (3–9 years) with SM appear promising when using a combined systems/behavioural intervention. However, limited fully powered systematic evaluations and the inconsistent use of outcome measures only allow for tentative conclusions to be drawn. There is an urgent need for more fully powered experimental studies which use consistent approaches to outcome measurement and include more representative and diverse samples, with consideration of moderators of treatment outcomes and of treatment cost‐effectiveness. To achieve this, regional and international research collaborations and measurement harmonisation (National Quality Forum, 2010) are crucial. While an international consensus on standard sets of outcome measures has already been achieved in other areas of child mental health (e.g. Krause et al., 2021), a similar approach is needed for SM. This will ensure the most can be made of future research through data sharing between regional and international groups to enable far greater clarity about how to achieve optimal outcomes for children and adolescents with SM.

## AUTHOR CONTRIBUTIONS

**Gino Hipolito**: Conceptualization; Data curation; Formal analysis; Funding acquisition; Investigation; Methodology; Project administration; Software; Validation; Visualization; Writing–original draft; Writing–review & editing. **Emma Pagnamenta**: Conceptualization; Formal analysis; Methodology; Resources; Supervision; Validation; Writing–review & editing. **Helen Stacey**: Formal analysis; Validation; Writing–review & editing. **Emily Wright**: Formal analysis; Validation; Writing–review & editing. **Victoria Joffe**: Conceptualization; Methodology; Supervision; Writing–review & editing. **Kou Murayama**: Methodology; Supervision; Validation; Writing–review & editing. **Cathy Creswell**: Conceptualization; Funding acquisition; Methodology; Supervision; Validation; Visualization; Writing–review & editing.

## CONFLICT OF INTEREST STATEMENT

The authors have declared that they have no competing or potential conflicts of interest.

## ETHICAL CONSIDERATIONS

No ethical approval or patient consent was required for this research review.

## PERMISSION TO PRODUCE MATERIAL FROM OTHER SOURCES

Not presenting material from other sources apart from data extracted from journals, dissertations and requested from Danielle Cornacchio and Beate Oerbeck regarding their research.

## Supporting information

Supporting Information S1

## Data Availability

The data that support the findings of this study are available on request from the corresponding author.
