# Supplementary material for: A systematic review and meta‐analysis of nonpharmacological interventions for children and adolescents with selective mutism
Source: JCPP Adv. 2023 May 3;3(3):e12166. doi: 10.1002/jcv2.12166 (PMC10501694; doi:10.1002/jcv2.12166)
Supplement: Supplementary file 1 — Supporting Information S1 [file JCV2-3-e12166-s001.docx]

**A systematic review and meta-analysis of nonpharmacological interventions for children and adolescents with selective mutism**

**Supporting Information**

**Appendix S1. Search strategy, eligibility criteria, data extraction, selection & quality appraisal procedures**

The following databases were searched: Medline, EMBASE, PsychINFO, Web of Science, The Cochrane Controlled Trials Register, ICTRP (WHO), CINAHL, ProQuest Dissertation and Theses Global, Educational Resources Information Centre (ERIC), British Education Index, Education Research Complete, EMCARE and Child Development & Adolescent Studies. The subject terms and key words used in the searches can be found in **Table S1**.

Hand searches covered bibliographies of narrative reviews and systematic reviews since Stone et al. 2002 (Cohan, Chavira & Stein, 2006; Muris & Ollendick, 2015; Østergaard, 2018; Sharkey & McNicholas, 2008; Standart & LeCouteur, 2003; Viana, Beidel & Rabian, 2009; Wong, 2010 and Zakszeski & DuPaul, 2017) and bibliographies of key books on selective mutism (Cline & Baldwin, 2005; Johnson & Wintgens, 2016, Kotrba, 2014 and Smith & Sluckin, 2014). In order to identify recent papers that may not be accessible via databases, forward citation hand searches were performed during the search dates above for articles ‘cited by’ for two key RCTs, Bergman et al. (2013) and Oerbeck et al. (2014) using Google scholar. Hand searches were also performed on bibliographies of eligible papers identified through the searches.

*Study eligibility criteria*

Criteria for the inclusion of studies in the systematic review were:

1. The study is written in the English language.
2. The study is in the form of a paper, dissertation or book chapter.
3. Participants are between the ages of 0 – 18 years or have a mean age of 18 years or under.
4. Participants have a clearly stated diagnosis of selective mutism or elective mutism.
5. The study is an experimental, quasi experimental or pre-experimental design that primarily investigates nonpharmacological intervention(s). It may include pharmacological intervention as a comparator but not mixed with the non-pharmacological intervention. Participants may be taking medication if they have been on a stable dose for at least 1 month (4 weeks) prior to intervention and during the intervention.
6. The study uses the interventions to target the SM.
7. The study uses pre and post intervention outcome measure(s) evaluating speaking behaviour, anxiety, well-being, learning or access to learning.
8. The study was carried out after October 1992 (post Stone et al., 2002).
9. The study has 3 or more participants (Hedges et al., 2013).

*Data management and selection process*

References from each database search were imported to Endnote X9.2. Duplicates were identified and deleted. The references and abstracts were then exported into Rayyan, a web app for systematic reviews (Ouzzani et al., 2016). The references from the search strategy were screened independently by the first and second coder (GH, HS) to identify the studies that potentially met the criteria outlined above with reasons for exclusion. Full texts of the studies that potentially met the criteria were retrieved and further assessed for eligibility independently by the first and third coder (GH, EP) in the first search and first and second coder (GH, HS) in the updated search. Consensus was reached through discussion. Any disagreement over the eligibility of a study was resolved by the senior reviewer (CC). Neither of the reviewers were blind to the journal titles or to the study authors.

*Data extraction*

A data extraction form was used to collate the following information from the studies: study design, age of participants, number of participants, gender, onset and/or duration of SM, bilingualism, co-morbidity, who provided the intervention, where the intervention was carried out, the type of intervention, dosage and length of time, outcomes used, main results and effect sizes and/or the statistics to calculate the effect sizes (e.g. means, standard deviations, autocorrelation or raw data for Single Case Experimental Designs- SCED). The primary outcomes are measures of the change in speaking behaviour and whether the child or young person still has SM or not after the intervention. Data extraction was carried out by GH with 20% of studies being verified by HS for non-statistical data and KM for statistical data in order to reduce bias and reduce errors. Missing information pertinent to the review was obtained where possible by emailing the author(s) of the study with up to three email attempts. Three authors were contacted requesting missing data or clarification about their intervention, and two replied.

*Quality Appraisal Tool*

The Standard Quality Assessment Criteria for evaluating primary research papers from a variety of fields (Kmet et al. 2004) was used to appraise the internal validity and risk of bias of the included studies. This is a flexible quality appraisal tool which can assess randomised and non-randomised study designs which suits the broad range of studies included in this review. The tool consists of 14 questions covering the research objective and design (use of randomisation, blinding, comparison groups), subject selection and description, outcomes measures, statistics and analysis, control for confounding, reporting of results and conclusions. For all studies, each item was rated as:

1. No- study did not resolve this item
2. Partial- study addressed query partially
3. Yes- study addressed query sufficiently

An adapted crib sheet customised for selective mutism research was designed to support the judgements of the coders. Moreover, Criteria 7: “If interventional and blinding of subjects was possible was it reported?” was not included in the quality appraisal since this is not generally possible for trials of psychological interventions. The calculation of the summary score deviates from Kmet et al.’s (2004) suggestion of excluding items marked “n/a” from the total possible score. This decision was made to give weight to the potential biases intrinsic to non-randomised compared to randomised studies. Consequently, each summary score was calculated from a total possible score of 26, and this summary score was calculated into a quality percentage. Studies scored below 75% were considered to have a high risk of bias (low quality) while studies 75% and over were deemed to have a low risk of bias (high quality).

Authors one and four (GH and EW) independently assessed the risk of bias in the included studies during the first search and coders one and three (GH and EP) appraised the studies accepted in the second and third search. Any disagreement between the review authors over the risk of bias in the particular studies were resolved through discussion with the senior reviewer (CC).

**Table S1. Subject terms and key words used in searches**

| **Person** | **Condition** | **Intervention** |
| --- | --- | --- |
| child* | “selective mutism” | therap* |
| adolescen* | “elective mutism” | program* |
| “young pe*” |  | treat* |
| “young adult*” |  | interven* |
| youth* |  | CBT |
| teen* |  | teach* |
|  |  | support* |

**Table S2. Quality Assessment**

| ID | Study | Criteria 1: Question/ objective sufficiently described? | Criteria 2: Study design evident and appropriate? | Criteria 3:  Method of subject/ comparison group selection or source of information/ input variables described and appropriate? | Criteria 4: Subject (and comparison group, if applicable) characteristics sufficiently described? | Criteria 5:  If interventional and random allocation was possible, was it reported? | Criteria 6:  If interventional and blinding of investigators was possible, was it reported? | Criteria 8:  Outcome and (if applicable) exposure measure(s) well defined and robust to measurement/ misclassification bias? | Criteria 9: Sample size appropriate? | Criteria 10: Analytic methods described/ justified and appropriate? | Criteria 11: Some estimate of variance is reported for the main results? | Criteria 12: Controlled for confounding? | Criteria 13: Results reported in sufficient detail? | Criteria 14: Conclusions supported by the results? | Score out of 26 (%) | Risk of Bias |
| --- | --- | --- | --- | --- | --- | --- | --- | --- | --- | --- | --- | --- | --- | --- | --- | --- |
| 1 | Ooi et al. (2016) |  |  |  |  |  |  |  |  |  |  |  |  |  | 20 (76.9) | Low |
| 2 | Esposito et al. (2017) |  |  |  |  |  |  |  |  |  |  |  |  |  | 23 (88.5) | Low |
| 3 | Bergman et al. (2013) |  |  |  |  |  |  |  |  |  |  |  |  |  | 23 (88.5) | Low |
| 4 | Oerbeck et al. (2014) |  |  |  |  |  |  |  |  |  |  |  |  |  | 23 (88.5) | Low |
| 5 | Cornacchio et al. (2019) |  |  |  |  |  |  |  |  |  |  |  |  |  | 22 (84.6) | Low |
| 6 | Bunnell et al. (2018) |  |  |  |  |  |  |  |  |  |  |  |  |  | 22 (84.6) | Low |
| 7 | Stone (2000) |  |  |  |  |  |  |  |  |  |  |  |  |  | 18 (69.2) | High |
| 8 | Vecchio (2008); Vecchio & Kearney (2009) |  |  |  |  |  |  |  |  |  |  |  |  |  | 16 (61.5) | High |
| 9 | Mitchell & Kratochwill (2013) |  |  |  |  |  |  |  |  |  |  |  |  |  | 18 (69.2) | High |
| 10 | Solz (2015) |  |  |  |  |  |  |  |  |  |  |  |  |  | 14 (53.8) | High |
| 11 | Siroky (2019) |  |  |  |  |  |  |  |  |  |  |  |  |  | 17 (65.4) | High |
| 12 | Woodcock et al. (2007) |  |  |  |  |  |  |  |  |  |  |  |  |  | 6 (23.1) | High |
| 13 | Sharkey et al. (2008) |  |  |  |  |  |  |  |  |  |  |  |  |  | 14 (53.8) | High |
| 14 | Oerbeck et al. (2011) |  |  |  |  |  |  |  |  |  |  |  |  |  | 18 (69.2) | High |
| 15 | Oerbeck et al. (2015) |  |  |  |  |  |  |  |  |  |  |  |  |  | 18 (69.2) | High |
| 16 | Klein et al. (2017) |  |  |  |  |  |  |  |  |  |  |  |  |  | 22 (84.6) | Low |
| 17 | Oerbeck et al. (2018) |  |  |  |  |  |  |  |  |  |  |  |  |  | 20 (76.9) | Low |
| 18 | Aldrich et al. (2021) |  |  |  |  |  |  |  |  |  |  |  |  |  | 18 (69.2) | High |
| 19 | Tan et al. (2021) |  |  |  |  |  |  |  |  |  |  |  |  |  | 18 (69.2) | High |
| 20 | Ortega (2011) |  |  |  |  |  |  |  |  |  |  |  |  |  | 16 (61.5) | High |
| 21 | Paasivirta (2012) |  |  |  |  |  |  |  |  |  |  |  |  |  | 15 (57.7) | High |
| 22 | Roslin (2013) |  |  |  |  |  |  |  |  |  |  |  |  |  | 15 (57.7) | High |
| 23 | Bunnell et al. (2016) |  |  |  |  |  |  |  |  |  |  |  |  |  | 10 (38.5) | High |
| 24 | Bork (2016); Bork and Bennett (2020) |  |  |  |  |  |  |  |  |  |  |  |  |  | 10 (38.5) | High |
| 25 | Haggerty (2020) |  |  |  |  |  |  |  |  |  |  |  |  |  | 16 (61.5) | High |
| YES PARTIAL NO | | | | | | | | | | | | | | | | |

Scores greater than 75% are considered to be low risk of bias.

Scores less than 75% are considered to be high risk of bias.

| **Table S3. Outcomes for selective mutism intervention studies** |
| --- |

| ID | Authors | Speaking Behaviour Hedges’ g effect size | Anxiety Hedges’ g effect size | SM remission | Risk of Bias | Limitations |
| --- | --- | --- | --- | --- | --- | --- |

| ***Experimental: Randomised Control Trial (RCT) vs. Active Control*** |
| --- |

| 1 | Ooi et al. (2016) | *Tx vs Active SMQ ES = -0.05 (no effect), SE: 0.44, 95% CI: -0.91 to 0.81. ***SMQ ES = 0.30 (small), SE: 0.30, 95% CI: -0.29 to 0.89. | *Tx vs Active ACAS (parent) ES = 0.59 (medium increase), SE: 0.45, 95% CI: -0.28 to 1.46. *Tx vs Active ACAS (child) ES = 0.21 (small increase), SE: 0.44, 95% CI: -0.64 to 1.08.  ***ACAS (parent) ES = 0.34 (small increase), SE: 0.30, 95% CI: -0.25 to 0.93.  ***ACAS (child) ES = -0.15 (negligible), SE: 0.29, 95% CI: -0.73 to 0.43. | NR | Low | Sample size,  Blinding,  No control for confounding ('as treated' analysis). |
| --- | --- | --- | --- | --- | --- | --- |
| 2 | Esposito et al. (2017) | *Tx vs Active SMQ ES = 1.00 (large), SE: 0.18, 95% CI: 0.65 to 1.36.  ***SMQ ES = 0.90 (large), SE: 0.13, 95% CI: 0.64 to 1.16. | *Tx vs Active CBCL Anxiety/Depression (parent) ES = -0.96 (large), SE: 0.18, 95% CI: -1.32 to -0.61. ***CBCL Anxiety (parent) ES = -0.58 (medium), SE: 0.13, 95% CI: -0.84 to -0.32. | NR | Low | Analytic methods (no effect sizes),  No control for confounding ('per protocol'). |
|  | ***Experimental: RCT vs. Waiting List (WL)*** | | | | | |
| 3 | Bergman et al. (2013) | *Tx vs WL SMQ ES at mid-treatment = 0.77 (medium), SE: 0.46, 95% CI: -0.12 to 1.67. *Tx vs WL SSQ ES at mid treatment = 1.18 (large), SE: 0.48, 95% CI: 0.24 to 2.12.  ***SMQ ES at 3 month follow up = 2.33 (large), SE: 0.55, 95% CI: 1.26 to 3.40. ***SSQ ES at 3 month follow up = 1.41 (large), SE: 0.39, 95%CI: 0.64 to 2.18. | *Tx vs WL SASC (parent) ES = -0.29 (small), SE: 0.44, 95% CI: -1.16 to 0.58. *Tx vs WL SASC (teacher) ES = -0.24 (small), SE: 0.44, 95% CI: -1.1 to 0.63.  ***SASC (parent) ES = -1.03 (large), SE: 0.34, 95%CI: -1.70 to -0.36.  ***SASC (teacher) ES = -0.36 (small), SE: 0.28, 95%CI: -0.91 to 0.19. | Remission 67% (14 out of 21) post treatment. **Risk Ratio (Treatment vs. WL controls) = 5.38, 95% CI: 0.31 to 92.73.** | Low | Sample size,  Risk of selection bias, Could not compare end of treatment results with control. |
| 4 | Oerbeck et al. (2014) | *Tx vs WL SMQ ES = 1.39 (large), SE: 0.45, 95% CI: 0.5 to 2.28.  *Tx vs WL SSQ ES = 0.92 (large), SE: 0.43, 95% CI: 0.08 to 1.76.  ***SMQ ES = 1.46 (large), SE : 0.40, 95% CI : 0.67 to 2.25. | NR | NR | Low | Sample size,  Blinding. |
| 5 | Cornacchio et al. (2019) | *Tx vs WL SMQ ES = 1.03 (large), SE: 0.41, 95% CI: 0.23 to 1.84.  ***SMQ ES at follow up = 1.19 (large), SE: 0.25, 95% CI: 0.71 to 1.67.  ***SSQ ES at follow up = 0.70 (medium), SE: 0.20, 95% CI: 0.30 to 1.10. | ***CBCL Anxiety (parent) ES = -0.27 (small) (converted from d) | Remission 18.5% (5 out of 27) post treatment, 54% (13 out of 24) after 14-week follow up. **Risk Ratio (Treatment vs. WL controls) = 3.20, 95% CI: 0.14 to 72.62.** | Low | Sample size,  Risk of selection bias. |
|  | ***Experimental: RCT comparing adapted treatments*** | | | | | |
| 6 | Bunnell et al. (2018) | N/A. Only one data point for speaking behaviour (Minutes to complete shaping hierarchy). | Anxiety data collected baseline and during sessions.  Session 1 ***iBT HRV ES = -0.09 (negligible), 95% CI: -1.34 to 1.15). tBT HRV ES = -0.19 (negligible), 95% CI: -1.43 to 1.05) rBT HRV ES = -0.21 (small), 95% CI: -1.45 to 1.04).  Session 2 ***iBT HRV ES = -0.15 (negligible), 95% CI: -1.39 to 1.09). tBT HRV ES = -0.08 (negligible), 95% CI: -1.32 to 1.16). rBT HRV ES = -0.15 (negligible), 95% CI: -1.39 to 1.09).  Session 1 ***iBT EDA ES = 0.50 (medium increase), 95% CI: -0.76 to 1.76). tBT EDA ES = 0.45 (medium increase), 95% CI: -0.81 to 1.70) rBT EDA ES = 1.49 (large increase), 95% CI: 0.09 to 2.90).  Session 2 *** iBT EDA ES = 1.33 (large increase), 95% CI: -0.04 to 2.70). tBT EDA ES = 0.85 (large increase), 95% CI: -0.44 to 2.15). rBT EDA ES = 1.44 (large increase), 95% CI: 0.05 to 2.83). | NR | Low | Sample size,  Risk of selection bias. |
|  | ***Experimental: Single Case Experimental Design (SCED)*** | | | | | |
| 7 | Stone (2000) | Videotape Training ***Total Words Spoken ES = 1.13 (large), SE: 0.62, 95% CI: -0.09 to 2.35. Self-modeling *** Total Words Spoken ES = 0.71 (large), SE: 0.51, 95% CI: -0.29 to 1.71. | Video Training ***CBCL Internalizing (parent) ES = -0.71 (medium), SE: 0.95, 95% CI: -2.58 to 1.15. Video Training ***CBCL Internalizing (teacher) ES = -0.34 (small), SE: 0.93, 95% CI: -2.2 to 1.49. Self modeling ***CBCL internalizing (parent) ES = -0.37 (small), SE: 0.93, 95% CI: -2.20 to 1.46.  Self modeling ***CBCL internalizing (teacher) ES = 0.08 (negligible increase), SE: 1.25, 95% CI: -2.38 to 2.54. | NR | High | Sample size,  Risk of selection bias, Blinding,  No estimates of variance. |
| 8 | Vecchio (2008); Vecchio & Kearney (2009) | Exposure Treatment vs. Contingency Management:  **^Mean Words Spoken per day (Child rated) ES = 0.74 (large) (converted from Cohen's d). **^Mean Words Spoken per day (Parent rated) ES = 0.36 (medium) (converted from Cohen's d).  **^Mean Words Spoken per day (Teacher rated) ES = 0.22 (small) (converted from Cohen's d). | ***CBCL internalizing (parent) ES = -0.48 (medium), SE: 0.61, 95% CI: -1.69 to 0.72. | NR | High | Sample size,  Risk of selection bias, Blinding,  Missing outcome measures (TRF, DRSB), No estimates of variance. |
| 9 | Mitchell & Kratochwill (2013) | **Words Per Minute ES = 2.08 (large), SE: 0.66, 95% CI: 0.78 to 3.38. | ***CBCL Anxiety (Parent) ES = -0.57 (medium), SE: 0.78, 95% CI: -2.1 to 0.96.  ***CBCL Anxiety (Teacher) ES = -0.13 (negligible), SE: 0.77, 95% CI: -1.63 to 1.38. | NR | High | Sample size,  Risk of selection bias, Non-randomised,  No control,  Analytic methods. |
| 10 | Solz (2015) | **Verbalisations ES = 0.05 (no effect), SE: 0.21, 95% CI: -0.36 to 0.46. **Meaningful Communication ES = 1.46 (large), SE: 0.29, 95% CI: 0.90 to 2.02. ***SMQ ES = 0.12 (negligible), SE: 0.80, 95% CI: -1.94 to 1.69. | Missing data for OSA and SUDS.  **SMACS ES = 0.44 (small increase), SE: 0.35, 95% CI: -0.25 to 1.13. | NR | High | Sample size,  Risk of selection bias, Non-randomised, Blinding,  No estimates of variance. |
| 11 | Siroky (2019) | **BROSB ES = 0.36 (small), SE: 0.27, 95% CI: -0.17 to 0.89.  ***SMQ ES = 2.12 (large), SE: 0.84, 95% CI: 0.47 to 3.77. | ***SASC-R (Parent) ES = -2.08 (large), SE: 0.76, 95% CI: -3.57 to -0.59.  ***SCARED (Parent) ES = -2.44 (large), SE: 0.86, 95% CI: -4.12 to -0.76. | Remission 60% (3 out of 5) post treatment. | High | Sample size,  Risk of selection bias, Non-randomised, Blinding,  No control for confounding. |
|  | ***Pre-Experimental: One-Group Pretest Postest Design*** | | | | | |
| 12 | Woodcock et al. (2007) | N/A. Lack of data to calculate effect size | NR | NR | High | Sample size,  Risk of selection bias, Non-randomised,  No control,  Blinding,  Analytic methods,  No estimate of variance, No control for confounding, Conclusions. |
| 13 | Sharkey et al. (2008) | ***SMQ ES = 1.36 (large)(converted from d) | ***SCAS (child) ES = -1.39 (large)(converted from d) | NR | High | Sample size,  Non-randomised,  No control,  Blinding,  No control for confounding. |
| 14 | Oerbeck et al. (2011) | ***SSQ ES = 4.11 (large), SE: 1.15, 95% CI: 1.86 to 6.36. | ***CBCL Anxiety (Parent) ES = 0.11 (negligible increase), SE: 0.36, 95% CI: -0.59 to 0.81.  ***CBCL Anxiety (Teacher) ES = -0.21 (small), SE: 0.36, 95% CI: -0.92 to 0.50. | NR | High | Sample size,  Non-randomised,  No control,  No control for confounding. |
| 15 | Oerbeck et al. (2015) | ***SMQ ES = 1.65 (large), SE: 0.31, 95% CI: 1.04 to 2.26.  ***SSQ ES = 1.19 (large), SE: 0.26, 95% CI: 0.68 to 1.70. | NR | Remission 50% (12 out of 24) 1 year follow up. | High | Sample size,  Non-randomised, Blinding,  No control for confounding. |
| 16 | Klein et al. (2017) | ***SMQ ES = 0.82 (large), SE: 0.18, 95% CI: 0.47 to 1.17. | ***CBCL Anxiety (Parent) ES = -0.41 (medium), SE: 0.16, 95% CI: -0.73 to -0.09.  ***CBCL Anxiety (Teacher) ES = -0.24 (small), SE: 0.16, 95% CI: -0.55 to 0.07. | NR | Low | Risk of selection bias, Non-randomised,  No control,  No control for confounding. |
| 17 | Oerbeck et al. (2018) | ***SMQ ES = 2.85 (large), SE: 0.41, 95% CI: 2.05 to 3.65.  ***SSQ ES = 1.90 (large), SE: 0.30, 95% CI: 1.31 to 2.49. | NR | Remission 70% (21 out of 30) 5 year follow up. | Low | Non-randomised, Blinding,  No control for confounding. |
| 18 | Aldrich et al. (2021) | ***SMQ ES = 0.70 (large), SE: 0.11, 95% CI: 0.47 to 0.93.  ***SSQ ES = 0.30 (small), SE: 0.14, 95% CI: 0.03 to 0.57 | ***SCAS (parent) ES = -0.08 (negligible), SE: 0.16, 95% CI: -0.39 to 0.23.  ***CALIS (child) ES = -0.48 (medium), SE: 0.12, 95% CI: -0.71 to -0.25.  ***CALIS (family) ES = -0.26 (small), SE: 0.12, 95% CI: -0.49 to -0.03. | NR | High | Non-randomised, Blinding,  Attrition in results (Analytical methods). |
| 19 | Tan et al. (2021) | ***SMQ ES = 0.49 (medium), SE: 0.23, 95% CI: 0.04 to 0.94. | ***SCAS (parent) ES = -0.26 (small), SE: 0.22, 95% CI: -0.69 to 0.17. | NR | High | Non-randomised, Blinding,  No control for confounding. |
|  | ***Pre-experimental: Single Subject Design*** | | | | | |
| 20 | Ortega (2011) | ***SMQ ES = 1.74 (large), SE: 0.90, 95% CI: -0.03 to 3.50. | NR | NR | High | Sample size,  Risk of selection bias, Non-randomised,  No control,  No control for confounding (single baseline data point weakens control for maturation). |
| 21 | Paasivirta (2012) | ***SMQ ES = 0.69 (medium), SE: 0.79, 95% CI: -0.86 to 2.24. | NR | NR | High | Sample size,  Risk of selection bias, Non-randomised,  No control,  Blinding,  No control for confounding. |
| 22 | Roslin (2013) | ***SMQ ES = 5.08 (large), SE: 1.38, 95% CI: 2.37 to 7.79. | NR | Remission 60% (3 out of 5) 1-3 month follow up. | High | Sample size,  Risk of selection bias, Non-randomised,  No control,  No estimates of variance,  No control of confounding. |
| 23 | Bunnell et al. (2016) | N/A lack of data to calculate effect sizes | N/A lack of data to calculate effect sizes | NR | High | Sample size,  Risk of selection bias, Non-randomised,  No control,  Analytic methods,  No estimate of variance, No control for confounding. |
| 24 | Bork (2016); Bork and Bennett (2020) | ***SMQ ES = 1.56 (large), SE: 1.06, 95% CI: -0.51 to 3.64. | ***Anxiety Scale (trained observer) ES = -0.38 (small), SE: 0.45, 95% CI: -1.26 to 0.50. | NR | High | Sample size,  Non-randomised,  No control,  Analytic methods,  No estimates of variance,  No control for confounding. |
| 25 | Haggerty (2020) | ***SMQ ES = 0.24 (small), SE: 0.20, 95% CI: -0.15 to 0.63. (calculated from pre- to posttx scores). | ***SCARED (Parent) ES = -0.34 (small), SE: 0.20, 95% CI: -0.73 to 0.05.  ***SCARED (Child) ES = -0.21 (small), SE: 0.20, 95% CI: -0.59 to 0.17. | NR | High | Sample size,  Risk of selection bias, Non-randomised,  No control,  No control for confounding. |
|  | NR- Not Reported; ES = Effect Size; SMQ = Selective Mutism Questionnaire; ACAS = Asian Children’s Anxiety Scale; CBCL = Child Behavior Checklist; SSQ = School Speech Questionnaire; SASC = Social Anxiety Scale for Children; iBT = mobile apps behavioral therapy; tBT = therapeutic tools Behavioral Therapy; rBT = reinforcement alone Behavioral Therapy; HRV = Heart Rate Variability; EDA = Electrodermal Activity; OSA- Observation Scale of Anxiety; SUDS- Subjective Units of Distress; SMACS = Selective Mutism and Anxiety Coding System; BROSB = Brief Rating of Observed Speaking Behavior; SCARED = Screen for Child Anxiety Related Disorders; SCAS = Spence Children’s Anxiety Scale; CALIS = Child Anxiety Life Interference Scale. | | | | | |
|  | Symbols: * Between subject effect size; ** Between subject SCED effect size using Hedges et al. (2013); **^ Between subject SCED effect size calculated in paper; *** Within case effect size | | | | | |

**Table S4. Study intervention components**

| Study | PsyEd | Rap | Exp | Rew | Cog | Cop | Psolv | ToC | Soc | PlayTx |
| --- | --- | --- | --- | --- | --- | --- | --- | --- | --- | --- |
| Ooi et al. 2016 |  |  |  |  |  |  |  |  |  |  |
| Esposito et al. 2017 |  |  |  |  |  |  |  |  |  |  |
| Bergman et al. 2013 |  |  |  |  |  |  |  |  |  |  |
| Oerbeck et al. 2014 |  |  |  |  |  |  |  |  |  |  |
| Cornacchio et al. 2019 |  |  |  |  |  |  |  |  |  |  |
| Bunnell et al. 2018 |  |  |  |  |  |  |  |  |  |  |
| Stone 2000 |  |  |  |  |  |  |  |  |  |  |
| Vecchio 2008; Vecchio & Kearney, 2009 |  |  |  |  |  |  |  |  |  |  |
| Mitchell & Kratochwill, 2013 |  |  |  |  |  |  |  |  |  |  |
| Solz 2015 |  |  |  |  |  |  |  |  |  |  |
| Siroky 2019 |  |  |  |  |  |  |  |  |  |  |
| Woodcock et al. 2007 |  |  |  |  |  |  |  |  |  |  |
| Sharkey et al. 2008 |  |  |  |  |  |  |  |  |  |  |
| Oerbeck et al. 2011 |  |  |  |  |  |  |  |  |  |  |
| Oerbeck et al. 2015 |  |  |  |  |  |  |  |  |  |  |
| Klein et al. 2017 |  |  |  |  |  |  |  |  |  |  |
| Oerbeck et al. 2018 |  |  |  |  |  |  |  |  |  |  |
| Aldrich et al. 2021 |  |  |  |  |  |  |  |  |  |  |
| Tan et al. 2021 |  |  |  |  |  |  |  |  |  |  |
| Ortega 2011 |  |  |  |  |  |  |  |  |  |  |
| Paasivirta 2012 |  |  |  |  |  |  |  |  |  |  |
| Roslin 2013 |  |  |  |  |  |  |  |  |  |  |
| Bunnell et al. 2016 |  |  |  |  |  |  |  |  |  |  |
| Bork 2016; Bork & Bennett 2020 |  |  |  |  |  |  |  |  |  |  |
| Haggerty 2020 |  |  |  |  |  |  |  |  |  |  |
| Total | 19 | 17 | 24 | 24 | 6 | 10 | 2 | 10 | 8 | 1 |
| PsyEd- Psycho-Education; Rap- Rapport building; Exp- Exposure; Rew- Reward System; Cog- Cognitive component; Cop- Coping Strategies; ProblSl- Problem Solving; ToC- Transfer of Control; Soc- Social Skills; PlayTx- Play Therapy | | | | | | | | | | |

**Table S5. Selective mutism intervention component definitions**

| **Psychoeducation (PsyEd)** is learning about SM and the treatment model, how SM affects the child or young person, and what helps and does not help the individual’s anxiety and ability to speak and participate. |
| --- |
| **Rapport building (Rap)** and/or **adapting interaction style** with individual with SM in order to make it easier for the child to speak. This could be designated rapport building time with clinician or teacher with child, or adapting interaction styles such as defocused communication or parent child interaction therapy. |
| **Exposure (Exp)** is when an individual experience the stimuli that they fear. In the context of SM, the individual is placed in a situation where there is an expectation to speak (e.g. stimulus fading, shaping, hierarchical exposure, prompting) or their voice is heard by people outside of their comfort zone (e.g. video modelling). (see Zakszeski and DuPaul (2017) for specific definitions for these behavioural strategies) |
| **Reward (Rew)** is positive reinforcement for appropriate behaviours (e.g. non-verbal communication, verbalisations and speaking) in the form of praise, tangible reinforcers (e.g. reward chart) or activity reinforcers (e.g. screen time). |
| **Cognitive (Cog)** component is where a specific part of the intervention explicitly focuses on a change in cognition or thinking, such as cognitive restructuring, testing out predictions based on anxious thoughts, modifying anxious self-talk into coping self-talk. |
| **Coping strategies (Cop)** strategies to help the individual with SM cope when feeling anxious including relaxation, emotional regulation, breathing exercises, and mindfulness. |
| **Problem solving (PSolv)** is equipping the child to actively solve problems linked to their SM (e.g. identifying their feelings and thoughts, then think of attitudes and actions that can help). |
| **Transfer of Control (ToC)** is the gradual transfer of knowledge, skills and methods from clinician to parent, teacher or child in order to produce long term therapeutic change (e.g. care-giver is given greater responsibility in selecting and implementing strategies outside of sessions). |
| **Social Skills Training (Soc)** is equipping the child with skills to improve interaction (e.g. learning phrases and sentences to initiate interaction with peers or asking teachers for help.) |
| **Play Therapy (PlayTx)** is helping children communicate and process emotions, thoughts and experiences through play in order to develop confidence and self-esteem, build relationships with others and alleviate anxiety. |

| **Table S6. Outcomes used in selective mutism intervention studies** | | | |  |
| --- | --- | --- | --- | --- |
| Outcomes measures used | Author of outcomes | Respondent | ***N***: Studies ID using outcome |  |
| ***Speaking behaviour outcome measures*** | | |  | |
| Selective Mutism Questionnaire (SMQ) | Bergman et al. (2008) | Parent | ***19***: 1-5, 10, 11, 13-22, 24, 25 | |
| School Speech Questionnaire (SSQ) | Bergman et al. (2002) | Teacher | ***12***: 3, 4, 5, 10, 14, 15, 17, 18, 20, 21, 22, 24 | |
| Daily Rating of Behaviors (DRB, DRCB, DRSB) | Vecchio & Kearney (2009) | Child, Parent, Teacher | ***3:*** 8, 10, 24 | |
| Minutes to complete speech hierarchy | Bunnell et al. (2016) | Clinician | ***2***: 6, 23 | |
| Selective Mutism Interaction Coding System (SMICS)*** | Kurtz, Comer & Masty (2007) | Independent Observer | ***2:*** 20, 22 | |
| Words spoken per observation | Stone (2000) | Independent Observer | ***1:*** 7 | |
| Type of verbal communication per observation | Stone (2000) | Parent, Teacher, Independent Observer | ***1:*** 7 | |
| No. words spoken by child per minute | Mitchell & Kratochwill (2013) | Parent, Teacher | ***1:*** 9 | |
| Revised Behavioural Observation Code for Selective Mutism (RBOCSM) | Sheridan & Kratochwill (1986) | Independent Observers | ***1:*** 9 | |
| Selective Mutism and Anxiety Coding System (SMACS) | Solz (2015) | Independent Observer | ***1:*** 10 | |
| Verbalisation frequency | Solz (2015) | Independent Observer | ***1:*** 10 | |
| Brief Rating of Observed Speaking Behavior (BROSB)** | Siroky (2019) | Parent | ***1:*** 11 | |
| Number of words spoken during activity | Siroky (2019) | Clinician | ***1:*** 11 | |
| Speaking pattern in various school situations | Woodcock et al. (2007) | NR | ***1:*** 12 | |
| Communication Rating Scale | Johnson & Wintgens (2001) | Child | ***1:*** 13 | |
| Verbal Communication (10-point likert scale) | Sharkey et al. (2008) | Clinician | ***1:*** 13 | |
| Non-Verbal Communication (10-point likert scale) | Sharkey et al. (2008) | Clinician | ***1:*** 13 | |
| Confident Speaking (10-point likert scale) | Sharkey et al. (2008) | Clinician | ***1:*** 13 | |
| Observational Record of Speech (4 levels: non-verbal; whispering; low volume and typical volume speech) | Aldrich et al. (2021) | Clinician, parents | ***1:*** 18 | |
| Frequency counts of verbal and non-verbal speech | Paasivirta (2012) | Independent Observer | ***1:*** 21 | |
| Number of new people spoken to in session | Bunnell et al. (2016) | Clinician | ***1:*** 23 | |
| Observed Speaking Behaviors from 5 daily exposure activities (no. spontaneous and responsive words, words per minute) | Haggerty (2020) | Independent Observer | ***1:*** 25 | |
|  |  |  |  | |
| ***Remission outcome measures*** |  |  |  | |
| The Anxiety Disorders Interview Schedule for Children for DSM-IV (ADIS-C/P) | Silverman & Albano (1996) | Independent Observer/Clinician with child/parent | ***6:*** 3, 5, 11, 15, 17, 22 | |
| Schedule for affective disorders and schizophrenia for school-aged children: present and lifetime version (K-SADS-PL) | Kaufman et al. (1997) | Clinician with child/parent | ***2:*** 15, 17 | |
|  |  |  |  | |
| ***Anxiety outcome measures*** | | |  | |
| Anxiety/Depression in CBCL | Achenbach & Rescorla (2001) | Parent | ***5:*** 2, 5, 9, 14, 16 | |
| Spence Children’s Anxiety Scale (SCAS) | Spence (1997) | Child | ***3:*** 13, 18, 19 | |
| Subjective Units of Distress (SUDS) | Bergman (2013) | Child, Parent | ***3:*** 6, 10, 23 | |
| Social Anxiety Scale for Children Revised (SASC-R) | La Greca & Stone (1993) | Parent, Teachers | ***2:*** 3, 11 | |
| Screen for Child Anxiety Related Disorders (SCARED) | Birmaher et al. (1999) | Parent | ***2:*** 11, 25 | |
| Asian Children’s Anxiety Scale (ACAS-C/P) | Koh et al. (2002) | Child, Parent | ***1:*** 1 | |
| Heart Rate Variability (HRV) | Mindware Technologies, LTD (2009) | Child | ***1:*** 6 | |
| Electrodermal Activity (EDA) | Mindware Technologies, LTD (2009) | Child | ***1:*** 6 | |
| Revised Children’s Manifest Anxiety Scale (RCMAS) | Reynolds & Richmond (1985) | Child | ***1:*** 7 | |
| Daily Rating of Anxiety (DRA, DRCA, DRSA) | Vecchio & Kearney (2009) | Child, Parent, Teacher | ***1:*** 8 | |
| Observation Scale of Anxiety (OSA) | Solz (2015) | Independent Observer | ***1:*** 10 | |
| Selective Mutism and Anxiety Coding System (SMACS) | Solz (2015) | Independent Observer | ***1:*** 10 | |
| Fear of Negative Evaluation (FNE) subscale of SASC-R | La Greca & Stone (1993) | Parent | ***1:*** 11 | |
| Separation Anxiety (10-point likert scale) | Sharkey et al. (2008) | Clinician | ***1:*** 13 | |
| Speaking levels in school setting (6 levels) | Oerbeck et al. (2011) | NR | ***1:*** 14 | |
| Observed anxiety Levels (4 levels) | Bork (2016) | Independent Observer | ***1:*** 24 | |
| Daily Behavior Report (DBR) based on 3 highest items on the SCARED | Haggerty (2020) | Parents | ***1:*** 25 | |
|  |  |  |  | |
| ***Condition severity and improvement outcome measures*** | | |  | |
| Clinical Global Impression Scale (CGI) Severity/Improvement | Guy & Bonato (1970) | Clinician, parent | ***7:*** 1, 3, 5, 13, 14, 15, 19 | |
| Clinical Severity Rating (CSR) as part of the ADIS | Silverman & Albano (1996) | Clinician | ***3:*** 3, 5, 11, | |
| Goal Attainment Scaling (GAS) | Kiresuk, Smith & Cardillo (1994) | Parent, Teacher | **2:** 7, 9 | |
| Severity of Behaviour Form* | Mitchell & Kratochwill (2013) | Parent, Teacher | ***1:*** 9 | |
|  |  |  |  | |
| ***Broader life outcome measures*** |  |  |  | |
| Child Behavior Checklist (CBCL) | Achenbach & Rescorla (2001) | Parent | ***6:*** 2, 7, 8, 9, 14, 19 | |
| Teacher’s Report Form (TRF) | Achenbach & Rescorla (2001) | Teacher | ***4:*** 7, 8, 9, 14 | |
| Clinical Global Assessment Scale (CGAS) | Shaffer et al. (1983) | Clinician | ***3:*** 5, 13, 19 | |
| Child Anxiety Life Interference Scale (CALIS) | Lyneham et al. (2013) | Child, Parent | ***1:*** 18 | |
| Strong Narrative Assessment Procedure (SNAP) | Strong (1998) | Clinician | ***1:*** 3 | |
| Test of Narrative Language Comprehension and Oral Narration (TNL C/O) | Gillam & Pearson (2004) | Clinician | ***1:*** 16 | |

| Impairment Rating Scale (IRS), school/academic impairment | Fabiano et al. (2006) | Teacher | ***1:*** 5 |
| --- | --- | --- | --- |
| School Competence Scale in CBCL | Achenbach & Rescorla (2001) | Parent | ***1:*** 2 |

| Inventory of Life Quality in Children and Adolescents (ILC) -Norwegian version. | Jozefiak (2011) | Child | ***1:*** 17 |
| --- | --- | --- | --- |
|  |  |  |  |
| ***Change in adult interaction style*** |  |  |  |
| Selective Mutism Interaction Coding System (SMICS)*** | Kurtz, Comer & Masty (2007) | Independent Observer | ***2:*** 20, 22 |
| Change in Teacher-Child Interaction*** | Paasivirta (2012) | Independent Observer | ***1:*** 21 |
| Change in Parent-Child Interaction*** | Roslin (2013) | Independent Observer | ***1:*** 22 |
|  |  |  |  |
| *adapted from Parent Screening Questionnaire and School Screening Questionnaire (Black & Uhde, 1994); ** based on the SMQ; *** based on Dyadic Parent-Child Interaction Coding System (DPICS, Eyberg et al. 2004); NR- Not Reported; | | | |

**Appendix S2. Within case effect sizes for selective mutism outcomes and percentage of remission**

*Improvements in speaking behaviours (see* ***Table S3****).*

All studies that used the SMQ reported an increase in speaking behaviours in the home, school and social situations from pre to post treatment, with within-case effects ranging from slight to large; these included studies that examined applied behaviour therapy only (Hedges’ g: 0.12 to 1.56), combined behavioural and systems approaches (Hedges’ g: 0.24 to 5.08), and the psychomotor approach (Hedges’ g: 0.90, SE: 0.13, 95% CI: 0.64 to 1.16).

The SSQ was only used in studies that evaluated the combined behavioural and systems approach, but these studies indicated increased speaking behaviours at school with small to large within-case effect sizes (Hedges’ g: 0.30 to 4.11).

*SM remission*

Data on remission from SM was only presented in six studies evaluating the combined behavioural and systems approaches. Bergman et al. (2013) reported that 67% (14 out of 21) of children no longer met diagnostic criteria for SM at the end of treatment. Siroky (2019) used a condensed version of Bergman et al.’s (2013) approach and found similar rates of remission (60%, 3 out of 5). Roslin’s (2013) PCIT-SM also had similar rates of remission (60%, 3 out of 5) at follow up. In contrast, Cornacchio et al.’s (2019) intensive group version of PCIT-SM reported that only 18.5% (5 out of 27, attrition = 2) of children recovered from SM posttreatment and 54% (13 out of 24, attrition = 5) after 14-week follow up. Longer-term follow-up studies of Oerbeck et al. (2011) and Oerbeck et al. (2014) indicated that 50% (12 out of 24) of children who had received treatment no longer met DSM diagnostic criteria at a 1-year (Oerbeck et al., 2015), and 70% (21 out of 30, attrition = 2)) at a 5 year follow up (Oerbeck et al. 2018).

*Reductions in anxiety*

There were mixed results for anxiety outcomes. Three studies using behaviour therapy only ranged between a medium size reduction to a small increase in parent reported anxiety after the intervention (Hedges’ g = -0.48 to 0.34). Child reported anxiety in Ooi et al’s (2016) study indicated only a slight decrease after intervention (Hedges’ g = -0.15, SE: 0.29, 95% CI: -0.73 to 0.43).

Studies using a combined behavioural and systems approach also showed a range of effects from large decreases to slight increases in parental reported anxiety (Hedges g: -2.44 to 0.11). Teacher reported anxiety measures in the same studies also displayed inconsistencies from small decreases to slight increases (Hedges’ g: -0.36 to 0.08). Two studies using child reported anxiety measures found small to large effects in decreasing anxiety after treatment (Hedges’ g = -1.39 to -0.21).

Esposito’s psychomotor approach was associated with a medium within-case reduction of parent reported anxiety (Hedges’ g = -0.58, SE: 0.13, 95% CI: -0.84 to -0.32).

*Remission of Social Anxiety Disorder (Social Phobia)*

In the studies that assessed for social anxiety disorder at the beginning and post treatment/follow up, there was, again, varied evidence of success. Remission of social anxiety disorder ranged from 66% (2 out of 3) to 94% (17 out of 18) immediately post treatment (Bergman et al. 2013; Siroky 2019). Remission rates in other studies at longer term follow up assessments displayed steady improvement over time with 21% (5 out of 24) remission after 1 year which then increased to 77% (23 out of 30, attrition = 2) at 5 year follow up (Oerbeck et al. 2015; Oerbeck et al. 2018). Cornacchio et al. (2019) assessed whether children met social anxiety disorder criteria at baseline (72%, 21 out of 29), posttreatment (63%, 17 out of 27, attrition = 2) and 14-week follow up (58%, 14 out of 24, attrition = 5) indicating a downward trend after intervention but it was not clear if those diagnosed at baseline were in the attrition group.

*Well-being, Interference and Quality of Life*

Tan and colleagues’ (2021) behavioural based virtual reality exposure therapy (VRET) showed a small within case effect size in reducing CBCL total problems (Hedges’g = -0.35, SE: 0.22, 95% CI: -0.79 to 0.09). Stone’s (2000) Videotape Training Intervention was associated with a reduction in parent/carer reported CBCL total problems with a small within case effect size (Hedges’ g = -0.26, SE: 0.43, 95% CI: -1.11 to 0.59) while the Self-Modelling Intervention had a small within case effect size (Hedges’ g = -0.34, SE: 0.44, 95% CI: -1.21 to 0.53). Esposito and colleagues (2017) reported a reduction in CBCL total problems with a large within case effect size following Psychomotor therapy (Hedges’ g = -0.98, SE: 0.15, 95% CI: -1.27 to -0.69).

Aldrich et al’s (2021) study measured the interference of anxiety in daily activities for the child and family and found the multidisciplinary SM treatment group reduced the impairment in daily functioning with a medium (Hedges’ g = -0.48, SE: 0.12, 95% CI: -0.71 to-0.25) and small (Hedges’ g = -0.26, SE: 0.12, 95% CI: -0.49 to -0.03) within case effect size respectively. Three studies (Cornacchio et al. 2019; Sharkey et al. 2008; Tan et al. 2021) used the CGAS outcomes. The two combined behavioural and systems approach studies showed improved functioning at home, school and with peers with large effect sizes (Hedges’ g = 1.28 to 2.88); while Tan et al.’s (2021) behavioural approach improved functioning with a medium effect size (Hedges’ g = 0.49, SE: 0.23, 95% CI: 0.04 to 0.94).

Oerbeck et al. (2018) was the only study that measured quality of life. The study did not have pre- and post- quality of life measures but instead compared the children’s post 5-year intervention results with Norwegian school children of the same age and found no significant difference between their Inventory of Life Quality in Children and Adolescents (ILC) scores (p = .82).

*Academic impairment*

Cornacchio et al. (2019) reported a reduction in academic impairment with a large within case effect size (Hedges’ g = -1.32, SE: 0.25, 95% CI: -1.81 to -0.83) using the IRS following combined behavioural and systems approach. Some reduction was found following the combined behavioural, systems and psychomotor approach using the CBCL School Competence scale but this was not statistically significant with a small effect size (Hedges’ g = -0.33, SE: 0.12, 95% CI: -0.57 to -0.09).
